# Missense Mutation R338W in *ARHGEF9* in a Family with X-linked Intellectual Disability with Variable Macrocephaly and Macro-Orchidism

**DOI:** 10.3389/fnmol.2015.00083

**Published:** 2016-01-20

**Authors:** Philip Long, Melanie M. May, Victoria M. James, Simone Grannò, John P. Johnson, Patrick Tarpey, Roger E. Stevenson, Kirsten Harvey, Charles E. Schwartz, Robert J. Harvey

**Affiliations:** ^1^Department of Pharmacology, UCL School of PharmacyLondon, UK; ^2^JC Self Research Institute, Greenwood Genetic CenterGreenwood, SC, USA; ^3^Department of Medical Genetics, Shodair Children’s HospitalHelena, MT, USA; ^4^Wellcome Trust Sanger Institute, Wellcome Trust Genome CampusHinxton, UK

**Keywords:** *ARHGEF9*, collybistin, gephyrin, PH domain, XLID

## Abstract

Non-syndromal X-linked intellectual disability (NS-XLID) represents a broad group of clinical disorders in which ID is the only clinically consistent manifestation. Although in many cases either chromosomal linkage data or knowledge of the >100 existing XLID genes has assisted mutation discovery, the underlying cause of disease remains unresolved in many families. We report the resolution of a large family (K8010) with NS-XLID, with variable macrocephaly and macro-orchidism. Although a previous linkage study had mapped the locus to Xq12-q21, this region contained too many candidate genes to be analyzed using conventional approaches. However, X-chromosome exome sequencing, bioinformatics analysis and segregation analysis revealed a novel missense mutation (c.1012C>T; p.R338W) in *ARHGEF9*. This gene encodes collybistin (CB), a neuronal GDP-GTP exchange factor previously implicated in several cases of XLID, as well as clustering of gephyrin and GABA_A_ receptors at inhibitory synapses. Molecular modeling of the CB R338W substitution revealed that this change results in the substitution of a long electropositive side-chain with a large non-charged hydrophobic side-chain. The R338W change is predicted to result in clashes with adjacent amino acids (K363 and N335) and disruption of electrostatic potential and local folding of the PH domain, which is known to bind phosphatidylinositol-3-phosphate (PI_3_P/PtdIns-3-P). Consistent with this finding, functional assays revealed that recombinant CB CB2_SH3−_^R338W^ was deficient in PI_3_P binding and was not able to translocate EGFP-gephyrin to submembrane microaggregates in an *in vitro* clustering assay. Taken together, these results suggest that the R338W mutation in *ARHGEF9* is the underlying cause of NS-XLID in this family.

## Introduction

Intellectual disability (ID) is characterized by significantly impaired intellectual and adaptive function, and is often defined by an IQ score below 70 in addition to deficits in two or more adaptive behaviors (e.g., social skills, problem solving) that affect everyday life. ID is also subdivided into syndromal ID, where ID is associated with other clinical, morphological, or behavioral symptoms or non-syndromal ID, where intellectual deficits appear without other associated defects (Stevenson et al., 2012). X-linked intellectual disability (XLID) refers to forms of ID typically associated with X-linked recessive inheritance. Mutations in monogenic XLID have been reported in >100 genes, many of which are now used in routine diagnostic screening panels (Basehore et al., 2015). Despite screening for mutations in selected known XLID genes by conventional linkage/candidate gene analysis or array CGH for examining copy number variants (CNVs), large number of families mapping to the X-chromosome remained unresolved (Lubs et al., 2012). These cases either represent undiscovered disease-relevant mutations in known genes, or causal mutations in novel XLID loci that remain to be identified.

Mutation and gene discovery in XLID has recently been transformed by large-scale DNA sequencing approaches coupled with stringent variant filtering (Tarpey et al., 2009; Rauch et al., 2012; Gilissen et al., 2014; Redin et al., 2014; Hu et al., 2015; Niranjan et al., 2015; Tzschach et al., 2015). For example, a recent study in a large cohort of unresolved families with XLID revealed that 20% of families carried pathogenic variants in established XLID genes (Hu et al., 2015), as well as revealing seven novel XLID genes (*CLCN4, CNKSR2, FRMPD4, KLHL15, LAS1L, RLIM*, and *USP27X*) and two candidates (*CDK16* and *TAF1*). Another strategy, known as Affected Kindred/Cross-Cohort Analysis (Niranjan et al., 2015) has also identified variants in known and novel XLID genes including *PLXNA3, GRIPAP1, EphrinB1* and *OGT*. However, analysis of next-generation data sets has also highlighted a number of XLID genes where truncating variants or previously published “mutations” are observed at a relatively high frequency in normal controls, calling into question whether certain single nucleotide variants (SNVs) are indeed causal (Tarpey et al., 2009; Piton et al., 2013). This highlights the need for integrating structure-function based approaches into the analysis pipeline for validating potentially disease-causing variants.

In this study, we have combined next-generation sequencing, variant filtering and structure-function assays to resolve the cause of XLID in a large family (K8010) with NS-XLID, with variable macrocephaly and macro-orchidism (Johnson et al., 1998). The previous clinical study in this family revealed ten affected males and two affected females in two generations, as well as four obligatory carriers. Most affected males exhibited macrocephaly and macro-orchidism, which are typical signs of the fragile X syndrome. However, cytogenetic testing and analysis of *FMR1* indicated they did not have this syndrome. It was also notable that some normal males in the family also exhibited macro-orchidism and macrocephaly. Linkage analysis suggested that the causative gene was located on Xp11-q21 (Johnson et al., 1998). We present compelling evidence that the likely cause of XLID in this family is a missense mutation in *ARHGEF9*, encoding a neuronal RhoGEF known as collybistin (CB) involved in both inhibitory synaptic organization and mammalian target of rapamycin complex 1 (mTORC1) signaling pathways (Machado et al., 2015).

## Materials and Methods

### Subjects

Family K8010 was previously reported by Johnson et al. (1998). Briefly, the males resembled those with fragile X syndrome in that they had macrocephaly (abnormally large head, typically 2.5 standard deviations above normal for weight and gender), macro-orchidism (abnormally large testes), blue eyes, prominent jaw and long facies. Additionally, one carrier female was described as being “slow”. However, using linkage analysis, the locus was mapped to Xq12-q21 rather than Xq27.3. Additionally, *FMR1* gene analysis was negative (Johnson et al., 1998).

### Exon Capture and DNA Sequencing

Next generation sequencing was conducted as a partial follow-up of 15 probands from a large scale sequencing of 718 X-chromosome genes in 208 XLID probands (Tarpey et al., 2009). The deep sequencing was conducted using the Agilent SureSelect Human X chromosome kit (Takano et al., 2012). A novel and unique mutation in *ARHGEF9*, c.1012C>T, was noted in K8010. Segregation analysis was conducted using Sanger sequencing.

### Polymorphism Analysis

Screening of 566 normal individuals (420 males, 146 females) for the *ARHGEF9* c.1012C>T variant was carried out using allele-specific amplification. Primers used were: *ARHGEF9*-ASOF 5′-TACGGCCGCAACCAGCtGt 3′ and *ARHGEF9*-ASOR 5′-CCCATCAGTATTTGCCCACT-3′. The ASOF primer recognized the mutation, which is indicated by the “t” at the 3′ end of the primer. The third base from the 3′ end was also changed from an “a” to a “t” to increase the specificity of the PCR. The ASOR primer was designed so that the T_m_ of both primers were similar and to generate a PCR product of above 500 bp. Gradient duplex PCR analysis was conducted using mutation and normal samples to choose the optimum annealing temperature. High-throughput duplex PCR analysis with a mutation and normal sample as positive and negative controls.

### Molecular Modeling of the Collybistin R338W Mutation

The non-synonymous R338W substitution was modeled into the structure of rat CB (PDB 2DFK; Xiang et al., 2006) using the swapaa command in Chimera (Pettersen et al., 2004) using the Dunbrack backbone-dependent rotamer library (Dunbrack, 2002). This took into account the lowest clash score, highest number of H-bonds and highest rotamer probability. Electrostatic potential of wild-type and R338W mutant CB was calculated using the Adaptive Poisson-Boltzmann Solver (APBS) web server (Baker et al., 2001; http://www.poissonboltzmann.org/).

### Site-Directed Mutagenesis and Expression Constructs

Full-length human CB cDNAs were cloned into the vector pRK5 as previously described (Kalscheuer et al., 2009). Mutations were introduced into pRK5myc-hCB3_SH3−_ construct using the QuikChange site-directed mutagenesis kit (Agilent) and confirmed by Sanger DNA sequencing of the entire coding region.

### PI_3_P Pull-Down Assays

Human embryonic kidney (HEK293) cells were grown in DMEM supplemented with 10% (v/v) fetal bovine serum at 37°C, 5% CO_2_ and transfected with 4 μg pRK5myc-hCB3_SH3−_ wild-type or R338W mutants using FuGENE (Roche). After 24 h, transfected cells were solubilized in a buffer containing Triton X-100 (Sigma-Aldrich), 1%; 150 mM NaCl; 50 mM Tris, pH 7.4, with protease inhibitor cocktail (Roche, Sussex, UK). Insoluble material was removed by centrifugation at 16, 100× g for 20 min. Phosphatidylinositol-3-phosphate (PI_3_P/PtdIns-3-P) agarose beads (40 μl; Eschelon Biosciences) were incubated with cell lysates for 2 h at 4°C. Beads were washed four times in buffer. Proteins were eluted from beads by heating at 98°C for 3 min in 2 × sample loading buffer and then subjected to SDS-PAGE. Proteins binding to beads were detected by Western blotting using mouse anti-c-myc antibody (Sigma, 1:1000) and HRP-conjugated goat anti-mouse (Santa Cruz, 1:2000). Immunoreactivity was visualized using West Pico Chemiluminescent Substrate (Pierce). Expression levels of hCB3_SH3−_ and hCB3_SH3−_R338W, and PI_3_P pulldown assay results were assessed using an unpaired, two-tailed Student’s *t*-test.

### Gephyrin Clustering Assays

These were performed essentially as previously described (Harvey et al., 2004). HEK293 cells were co-transfected with pRK5myc-hCB3_SH3−_ wild-type, pRK5myc-hCB3_SH3+_ wild-type, pRK5myc-hCB3_SH3−_ R338W, pRK5myc-hCB3_SH3−_ R290H or pRK5myc-hCB3_SH3−_ R356N/R357N constructs at a 1:1 ratio with pEGFP-gephyrin using electroporation (Gene Pulser II, Bio-Rad). Cells were fixed after 24 h for 2 min in 4% (w/v) PFA in PBS. Immunostaining to detect CB was performed using a mouse anti-c-myc antibody (1:200, Sigma) and detected using an AlexaFluor 546 goat anti-mouse secondary antibody (1:600; Invitrogen). Counterstaining for cell nuclei was performed with DAPI (1:500; Life Technologies). Confocal microscopy was performed using a Zeiss LSM 710 META. All images were taken with a × 63 objective.

## Results

### Identification of a R338W Mutation in *ARHGEF9* in Family K8010

**Figure 1:**
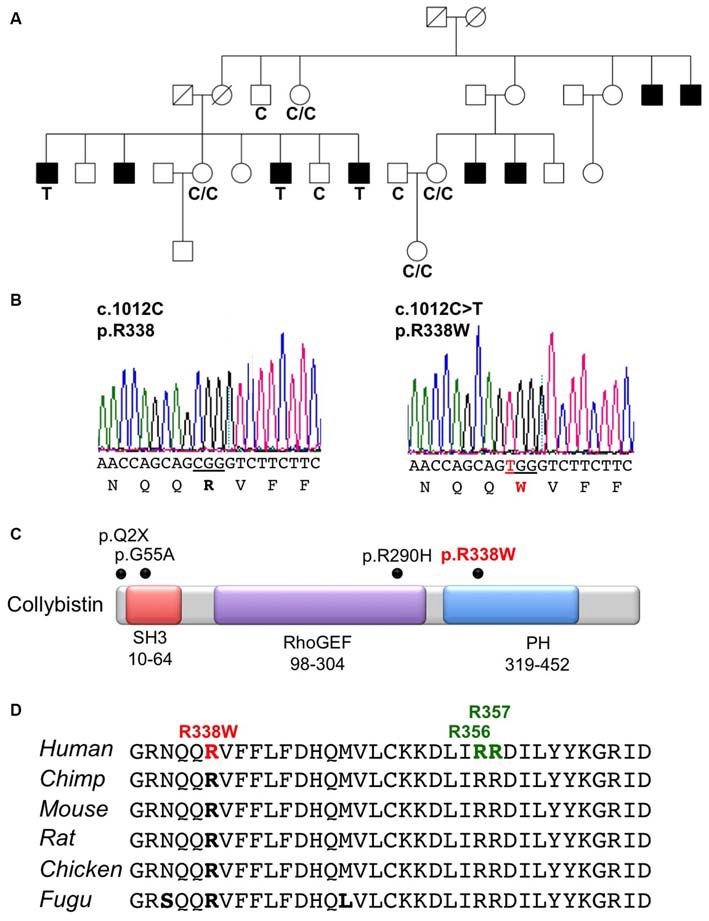
**Identification of a R338W mutation in *ARHGEF9* in family K8010. (A)** Pedigree of the K8010 family, which has been updated. Open symbols represent normal individuals, filled squares represent affected males. Individuals tested for the nucleotide substitution in each family are indicated with either a T (mutant allele) or a C (normal allele). **(B)** DNA sequence electropherograms for the c.1012C>T mutation reported in this study. **(C)** Schematic of the human collybistin (CB) protein with a regulatory SH3 domain, a catalytic RhoGEF domain and a pleckstrin homology (PH) domain. The relative locations of known missense and nonsense mutations in *ARHGEF9* are shown. **(D)** Sequence alignments of CB proteins from various species showing the high conservation of R338W in the PH domain. Note that R338W is not one of the known PI_3_P binding residues (R356 and R357, green).

X-chromosome exome sequencing of an individual male in family K8010 followed by bioinformatics analysis and filtering against publicly-available datasets revealed a novel missense change in *ARHGEF9*, chrX:62,885,810G>A, c.1012C>T; p.R338W, predicted as probably damaging (PolyPhen-2, score 1.000), damaging (SIFT) and a Combined Annotation Dependent Depletion (CADD) score of 19.37 (possibly pathogenic). This suggested that this missense mutation could be responsible for XLID, macrocephaly and macro-orchidism in this family. Subsequent segregation analysis using Sanger DNA sequencing indicated that the *ARHGEF9* c.1012C>T variant co-segregated with the phenotype in all individuals tested (Figures 1A,B).

### Collybistin Mutation R338W is Predicted to Disrupt PH Domain Folding

**Figure 2:**
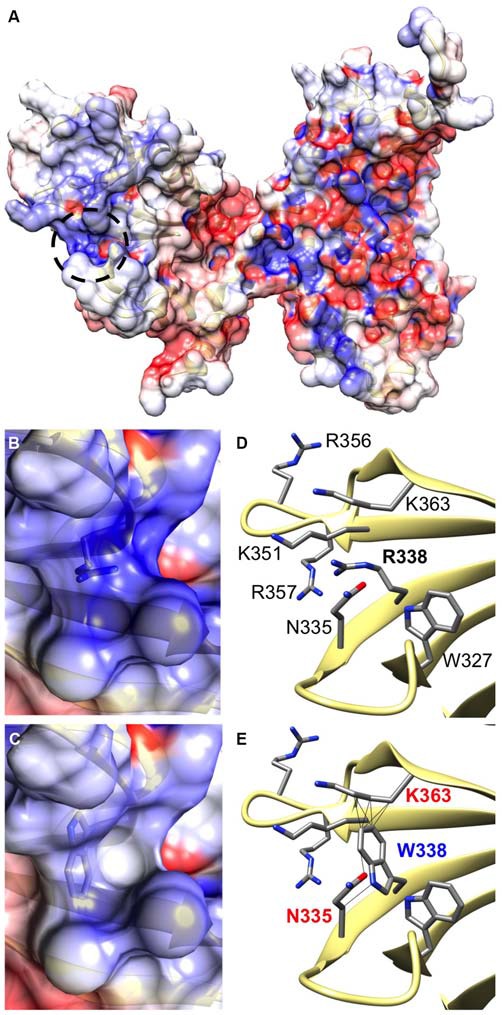
**The R338W substitution is predicted to influence the electrostatic potential and folding of the CB PH domain. (A)** Arginine 338 is located on the surface of the CB structure within the pleckstrin homology (PH) domain (circled by a black dotted line). It resides within a cleft between turns of β-sheet secondary structure **(D)**, in close proximity to other electropositive side-chains. Therefore, an area of hydrophobicity is created **(B)** close to the polar heads of the cell membrane. It is predicted that substitution of arginine with tryptophan at position 338 changes the hydrophobicity of this area, making it neutral in charge **(C)**. In addition, the bulky side-chain of tryptophan introduces clashes with surrounding side chains of lysine 363 and asparagine 335 **(E)**, disrupting the overall structure of the protein in this region.

CB belongs to the Dbl family of guanine nucleotide exchange factors, occurs in multiple splice variants (Kins et al., 2000; Harvey et al., 2004) and is specific for Cdc42, a small GTPase belonging to the Rho family (Xiang et al., 2006). CB has a multi-domain structure consisting of a regulatory SH3 domain, a catalytic RhoGEF domain and a pleckstrin homology (PH) domain (Figure 1C). Residue R338W is the first reported *ARHGEF9* missense mutation affecting a highly conserved residue in the PH domain (Figure 1D). Previous nonsense and missense mutations in CB have affected residues in the N-terminus (p.Q2X; Shimojima et al., 2011), regulatory SH3 domain (p.G55A; Harvey et al., 2004) and catalytic RhoGEF domain (p.R290H; Lemke et al., 2012; Papadopoulos et al., 2015). To assess how the R338W substitution might disrupt CB function, molecular modeling was performed using the structure of rat CB (PDB 2DFK; Xiang et al., 2006), which has a sequence identity of 85.5% to human CB [aligned using HHalign algorithm (Söding, 2005) within Clustal-Omega (Sievers et al., 2011)]. The R338W change replaces a long, electropositive side-chain (arginine) with a large non-charged, hydrophobic side-chain (tryptophan). Positively-charged residues are thought to be critical for interaction of the PH domain with the membrane (Xiang et al., 2006) and R338W clearly changes the electrostatic potential of the PH domain, as visualized using APBS (Figures 2A–C). R338W is also predicted to introduce a number of clashes with surrounding residues (e.g., N335, K363; Figures 2D,E), which is also predicted to affect interactions with membrane and the fold of the PH domain.

### Mutation R338W in Collybistin Disrupts Phosphatidylinositol-3-Phosphate Binding

**Figure 3:**
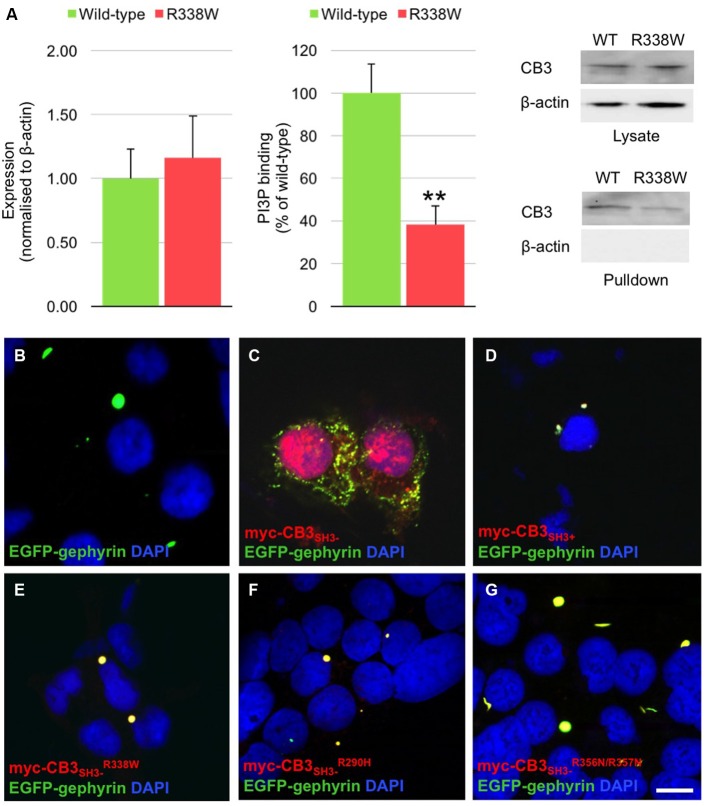
**R338W reduces collybistin PI_3_P binding and prevents formation of collybistin-mediated submembrane gephyrin clusters in cellular assays. (A)** Human embryonic kidney (HEK293) cells were transfected with plasmids encoding myc-CB3_SH3−_ or myc-CB3_SH3−_^R338W^ and cell lysates incubated with PI_3_P-conjugated agarose beads. After washing, bound material was subjected to SDS-PAGE and analyzed by immunoblotting with an anti-myc antibody (Sigma). Notably, no significant difference was observed in total level of myc-CB3_SH3−_^R338W^ expression in comparison with wild-type CB3_SH3−_, using β-actin as a loading control (**A**, left and right panels, normalized to wild-type 1.00 ± 0.23 vs. R338W 1.16 ± 0.33; wild-type ± SEM, *n* = 5). myc-CB3_SH3−_^R338W^ binding to PI_3_P was significantly reduced (62.7% ± 3.9%) in comparison to wild-type CB3_SH3−_. When we assessed the PI_3_P pull-down fraction as a percentage of the raw input, we saw a significant difference between wild-type and the R338W variant (**A**, middle and right panels; wild-type 100 ± 13.5 vs. R338W 38 ± 8.6; pull-down fraction ± SEM, *n* = 5, **p < 0.006, unpaired student’s *t*-test). No β-actin immunoreactivity was observed in PI_3_P pull-down samples, confirming the validity of this assay. **(B–G)** HEK293 cells were co-transfected with EGFP-gephyrin alone **(B)**, or with myc-CB3_SH3−_ **(C)**, myc-CB3_SH3+_ **(D)**, myc-CB3_SH3−_^R338W^ **(E)**, or the PI_3_P binding defective mutants myc-CB3_SH3−_^R290H^ **(F)**, and myc-CB3_SH3−_^R356N/R357N^ **(G)**. Cells were immunostained using anti-myc and AlexaFluor 546 antibodies and co-stained with a nuclear marker (DAPI). Note that while myc-CB3_SH3−_ co-localizes with gephyrin in submembrane clusters, all other CB variants co-localize with gephyrin in large intracellular aggregates, consistent with a lack of CB-mediated gephyrin clustering activity. Scale bar = 10 μm. **(C)** is a Z-projection, all other images are single plane.

The CB PH domain has previously been shown to play a key role in binding PI_3_P/PtdIns-3-P, a phosphoinositide with an emerging role in membrane trafficking and signal transduction. Deletion of the CB PH domain, or mutation of two key arginine residues (R356/R357) involved in PI_3_P binding has been demonstrated to abolish CB-mediated gephyrin clustering in functional assays (Harvey et al., 2004; Kalscheuer et al., 2009; Reddy-Alla et al., 2010). In order to determine whether the R338W mutation affected CB binding to PI_3_P, we performed pulldown assays using PI_3_P immobilized on agarose beads incubated with lysates of HEK293 cells transfected with either wild-type CB variant CB3_SH3−_ or mutant CB3_SH3−_^R338W^. Total expression of CB3_SH3−_^R338W^ was not significantly different to wild-type CB3_SH3−_ when normalized to β-actin expression (Figure 3A, left and right panels, wild-type CB2_SH3−_ 1.00 ± 0.23 vs. R338W 1.16 ± 0.33; normalized to wild-type ± SEM, *n* = 5). However, when the PI_3_P pull-down fraction was expressed as a percentage of raw input, a significant reduction of PI_3_P binding was observed for the R338W variant (Figure 3A, middle and right panels, wild-type 100 ± 13.5 vs. R338W 38 ± 8.6; pull-down fraction ± SEM, *n* = 5, *p* < 0.006).

### Mutation R338W Disrupts Collybistin-Mediated Gephyrin Clustering

Given the key role of CB in mediating gephyrin clustering at inhibitory synapses, we also investigated whether the R338W substitution affected the ability of CB to translocate gephyrin to submembrane microaggregates in a cellular clustering assay (Harvey et al., 2004; Kalscheuer et al., 2009). This involved co-expression of myc-tagged human CB (myc-CB3_SH3−_; Kalscheuer et al., 2009) with EGFP-gephyrin in HEK293 cells. CB variants containing the regulatory SH3 domain (CB3_SH3+_) typically co-localize with EGFP-gephyrin in large intracellular aggregates (Figures 3B,D; Kins et al., 2000; Harvey et al., 2004; Kalscheuer et al., 2009) and require neuroligins, GABA_A_R α2 or the small Rho-like GTPase TC10 for activation (Poulopoulos et al., 2009; Saiepour et al., 2010; Mayer et al., 2013). However, variants lacking the regulatory SH3 domain (e.g., CB3_SH3−_) typically result in the formation of EGFP-gephyrin submembrane clusters (Figure 3C). However, the CB3_SH3−_^R338W^ variant did not result in the formation of submembrane microaggregates with EGFP-gephyrin, but rather co-localized with EGFP-gephyrin in large intracellular aggregates (Figure 3E) similar to the distribution previously observed for CB3_SH3+_ or CB mutants that disrupt PI_3_P binding, such as R290H and the double mutant R356N/R357N (Figures 3F,G; Reddy-Alla et al., 2010; Papadopoulos et al., 2015). This demonstrates that the R338W substitution disrupts CB-mediated accumulation of gephyrin in submembrane microclusters.

## Discussion

This study reports the identification and functional characterization of a novel mutation (p.R338W) in *ARHGEF9* that is likely to represent the cause of XLID in family K8010. Using next-generation X-exome sequencing, Affected Kindred/Cross-Cohort Analysis and inheritance testing, we found a novel SNV (c.1012C>T; p.R338W) in *ARHGEF9* that segregated with the disease phenotype. Using molecular modeling and functional assays for CB PI_3_P binding and gephyrin clustering, we were able to establish the likely pathomechanism for p.R338W: a local disruption in the PH domain structure, leading to a reduction in PI_3_P binding and/or PH domain folding, and consequent loss in the ability of CB to mediate gephyrin clustering in an *in vitro* assay. The identification of *ARHGEF9* as the causative gene for family K8010 is consistent with previous studies that have identified CB as a neuronally-expressed RhoGEF with a key role in inhibitory synaptic transmission (Kins et al., 2000; Harvey et al., 2004). At selected inhibitory synapses, CB interacts with gephyrin (Kins et al., 2000; Harvey et al., 2004), a scaffolding protein with dual roles in inhibitory receptor clustering and molybdenum co-factor synthesis (Feng et al., 1998). CB knockout mice show increased anxiety and impaired spatial learning associated with a selective loss of GABA_A_Rs in the basolateral amygdala and hippocampus (Papadopoulos et al., 2007). Unsurprisingly, loss of CB clearly leads to significant changes in GABAergic inhibition, network excitability and synaptic plasticity (Jedlicka et al., 2009). Recent studies have also implicated CB in mTOR signaling: CB physically interacts with mTOR and inhibits mTORC1 signaling pathway and protein synthesis (Machado et al., 2015). This suggests that disruption of mTORC1 signaling pathways could also contribute to ID in patients with *ARHGEF9* loss-of-function mutations.

A number of mutations in *ARHGEF9* have been identified in patients encompassing missense and nonsense mutations, deletions and complex rearrangements (Table 1). Curiously, the associated phenotypes vary quite substantially. For example, Harvey et al. (2004) reported a p.G55A missense mutation in *ARHGEF9* associated with hyperekplexia, early infantile epileptic encephalopathy and severe psychomotor retardation (p.G55A, SH3 domain). By contrast, Shimojima et al. (2011) identified an *ARHGEF9* nonsense mutation (p.Q2X) in an individual with refractory seizures, right frontal polymicrogyria and severe psychomotor retardation. Lemke et al. (2012) also reported a p.R290H missense mutation in the CB RhoGEF domain associated with epilepsy and ID. Furthermore, large *de novo* deletions affecting *ARHGEF9* as well as neighboring genes *SPIN4* and *LOC92249* have been reported to be associated with complex phenotypes that include features such as partial seizures, delayed psychomotor development and generalized overgrowth (Table 1; Lesca et al., 2011; Shimojima et al., 2011). Lastly, a balanced chromosomal translocation (Kalscheuer et al., 2009) and a paracentric inversion (Marco et al., 2008) have been reported with yet more clinical features, including disturbed sleep-wake cycle, increased anxiety and aggressive behavior or hyperarousal, respectively.

**Table 1:** ***ARHGEF9* mutations and associated phenotypes**.

Mutation type	Nucleotide	Protein	Reported phenotype	Reference
Missense and nonsense mutations	c.4C>T	p.Q2X	Refractory seizures, right frontal polymicrogyria, severe psychomotor retardation, ataxia	Shimojima et al. (2011)
	c.869G>A	p.G55A	Hyperekplexia, early infantile epileptic encephalopathy and severe psychomotor retardation	Harvey et al. (2004)
	c.869G>A	p.R290H	Epilepsy and intellectual disability	Lemke et al. (2012)
	c.1012C>T	p.R338W	Intellectual disability with variable macrocephaly and macro-orchidism	This study
Deletions	*De novo* 737 kb deletion including *ARHGEF9, SPIN4, LOC92249*		Complex partial seizures, severely delayed psychomotor development, generalized overgrowth and trigonocephaly	Kalscheuer et al. (2009)
	*De novo* 1.29 Mb deletion of Xq11.11 including *ARHGEF9, SPIN4, LOC92249*		Delayed psychomotor development, loss of consciousness, hypotonia, cyanosis, generalized overgrowth, mild dysmorphic features, hyperactivity with attention deficit, limited social interaction	Marco et al. (2008)
Complex rearrangements	Balanced translocation 46, X, t(Xq11.1;18q11.21)		Disturbed sleep-wake cycle, late-onset epileptic seizures, increased anxiety, aggressive behavior and intellectual disability	Kalscheuer et al. (2009)
	Balanced *de novo* paracentric inversion (X)(q11.1;q27.3)		Hyperarousal (noise and social situations), global developmental delay, dysarthric speech, difficulty with smooth eye pursuit, bilateral lower extremity spasticity, brisk reflexes and extensor plantar responses, wide-based gait	Marco et al. (2008)

The exact reasons behind this clinical variability remains unknown, but are likely to be linked to several factors. Firstly, certain CB mutations (e.g., p.G55A, p.R290H, C-terminal truncations) clearly cause dominant-negative effects on gephyrin and GABA_A_ receptor clustering in neuronal systems (Harvey et al., 2004; Kalscheuer et al., 2009; Papadopoulos et al., 2015). Secondly, for female patients with CB mutations, the clinical features observed may depend on the degree of X-inactivation skewing. At least two studies involving a translocation or inversion in *ARHGEF9* have indicated skewed X inactivation in favor of the abnormal X chromosome (Marco et al., 2008; Kalscheuer et al., 2009). However, one emerging theme in functional studies of CB missense mutations appears to be loss of PI_3_P binding. Our own functional analysis suggests that the p.R338W variant causes a local disruption in the PH domain structure, leading to a reduction in PI_3_P binding and/or PH domain folding, and consequent loss in the ability of CB to mediate gephyrin clustering. Similar findings have recently been reported for the p.R290H mutation linked to epilepsy and ID, which appears to alter the strength of intramolecular interactions between the RhoGEF and PH domains, also leading to a loss of PI_3_P binding affinity (Papadopoulos et al., 2015). These results highlight the key role of phosphoinositide binding and correct localization of CB for synaptic function. However, given the variability in clinical phenotypes associated with CB mutations, it is also evident that next-generation sequencing diagnostics have a pivotal role to play in the diagnosis of X-linked disorders.

## Author Contributions

RJH and CES designed the experiments; JPJ contributed DNA samples; RES provided clinical evaluation; PL, SG, MMM, VMJ, PT and KH performed the experiments; CES, SG, PT, KH and RJH analyzed the data; CES and RJH wrote the paper. All authors were involved in revising the paper for important intellectual content, and gave final approval of the version to be published.

## Funding

This work was supported by the Medical Research Council (J004049 to RJH and KH), a NINDS grant (R01NS073854 to CES) and in part by the South Carolina Department of Disabilities and Special Needs (SC DDSN). The funders had no role in study design, data collection and analysis, decision to publish, or preparation of the manuscript. Dedicated to the memory of Ethan Francis Schwartz (1996–1998).

## Conflict of Interest Statement

The authors declare that the research was conducted in the absence of any commercial or financial relationships that could be construed as a potential conflict of interest.
